# Inhibition of Wild *Enterobacter cloacae* Biofilm Formation by Nanostructured Graphene- and Hexagonal Boron Nitride-Coated Surfaces

**DOI:** 10.3390/nano9010049

**Published:** 2019-01-02

**Authors:** Elsie Zurob, Geraldine Dennett, Dana Gentil, Francisco Montero-Silva, Ulrike Gerber, Pamela Naulín, Andrea Gómez, Raúl Fuentes, Sheila Lascano, Thiago Henrique Rodrigues da Cunha, Cristian Ramírez, Ricardo Henríquez, Valeria del Campo, Nelson Barrera, Marcela Wilkens, Carolina Parra

**Affiliations:** 1 Laboratorio Nanobiomateriales, Departamento de Física, Universidad Técnica Federico Santa María, Avenida España 1680, Valparaíso, Chile; elsie.zurob@usach.cl (E.Z.); g.dennett@gmail.com (G.D.); dana.gentil@usm.cl (D.G.); monteroster@gmail.com (F.M.-S.); 2 Laboratorio de Microbiología Básica y Aplicada, Universidad de Santiago de Chile, Avenida Libertador Bernardo O’Higgins 3363, Santiago, Chile; marcela.wilkens@usach.cl; 3 Faculty Environment and Natural Science, Institute of Biotechnology, Brandenburg University of Technology, Universitätsplatz 1, 01968 Senftenberg, Germany; gerberu@b-tu.de; 4 Facultad de Ciencias Biológicas, Pontificia Universidad Católica de Chile, Alameda 340, Santiago, Chile; pnaulin@uc.cl (P.N.); agomez@bio.puc.cl (A.G.); nbarrera@bio.puc.cl (N.B.); 5 Departamento de Industrias, Universidad Técnica Federico Santa María, Avenida España 1680, Valparaíso, Chile; raul.fuentes@usm.cl; 6 Departamento de Mecánica, Universidad Técnica Federico Santa María, Avda. Vicuña Mackenna 3939, Santiago, Chile; sheila.lascano@usm.cl; 7 Departamento de Física, CTNanotubos, Universidade Federal de Minas Gerais, Belo Horizonte 31310260, Brazil; thiago.cunha@ctnano.com.br; 8 Departamento de Ingeniería Química y Ambiental, Universidad Técnica Federico Santa María, Avenida España 1680, Valparaíso, Chile; cristian.ramirez@usm.cl; 9 Departamento de Física, Universidad Técnica Federico Santa María, Avenida España 1680, Valparaíso, Chile; ricardo.henriquez@usm.cl (R.H.); valeria.delcampo@usm.cl (V.d.C.)

**Keywords:** graphene, h-BN, nanostructured coatings, biofilms, *E. cloacae*

## Abstract

Although biofilm formation is a very effective mechanism to sustain bacterial life, it is detrimental in medical and industrial sectors. Current strategies to control biofilm proliferation are typically based on biocides, which exhibit a negative environmental impact. In the search for environmentally friendly solutions, nanotechnology opens the possibility to control the interaction between biological systems and colonized surfaces by introducing nanostructured coatings that have the potential to affect bacterial adhesion by modifying surface properties at the same scale. In this work, we present a study on the performance of graphene and hexagonal boron nitride coatings (h-BN) to reduce biofilm formation. In contraposition to planktonic state, we focused on evaluating the efficiency of graphene and h-BN at the irreversible stage of biofilm formation, where most of the biocide solutions have a poor performance. A wild *Enterobacter cloacae* strain was isolated, from fouling found in a natural environment, and used in these experiments. According to our results, graphene and h-BN coatings modify surface energy and electrostatic interactions with biological systems. This nanoscale modification determines a significant reduction in biofilm formation at its irreversible stage. No bactericidal effects were found, suggesting both coatings offer a biocompatible solution for biofilm and fouling control in a wide range of applications.

## 1. Introduction

Under natural conditions, microorganisms often encounter complex and hostile environments [1]. Their ability to quickly adapt to these changes in their surroundings will ensure their survival. The activation of survival mechanisms in bacteria relies on their ability to form communities called biofilms [2]. These mechanisms allow bacteria to attach to surfaces through the secretion of exopolymeric substances (EPS) [3], generating a three-dimensional enclosed matrix [4] composed mainly of polysaccharides, proteins, and DNA [5,6]. Biofilm formation provides bacteria with a defense against predators and chemical toxins; such as biocides and antibiotics [7].

Bacteria within biofilms are more resistant than those in planktonic or sessile state. Studies have shown that biofilm cells can tolerate up to 1000 times more antibiotic concentrations than their planktonic counterparts, and are even able to survive in environments exposed to biocides and UV radiation [8]. This makes it very hard to eradicate them once they have reached their biofilm form [9].

Although biofilm formation is a very effective mechanism for sustaining bacterial life, at the same time, it is unfavorable and harmful in human environments; such as in the medical field (where biofilms are responsible for at least 65% of all bacterial infections [8]), in food processing areas (where they lead to food spoilage [10]), and industrial sectors (where they increase fuel and energy consumption, and cause important economic losses [2,11,12]). In addition, biofilms have been linked to the proliferation of highly invasive freshwater microalgae such as *Didymosphenia geminate* (rock snot) [13].

Currently, these issues are addressed using biocides, which are chemical agents with antiseptic, disinfectant, or preservative properties used to control and prevent biofilm formation. The use of biocides does not only have an economic impact, but also is responsible for harmful by-products, many being toxic and even carcinogenic [14]. Biocides such as tributyltin (TBT), copper pyrithione (CuPT), triclosan [15], and quaternary ammonium compounds [16], have a severe impact on marine environments due to their high toxicity [11]. Chlorine is one of the most common antimicrobial agents used to control microorganisms, however, studies have shown that its efficiency applies mostly to planktonic bacteria causing a mild effect on biofilms [17].

In fact, it is important to highlight that a vast majority of studies regarding biofilm control and prevention have been performed on planktonic cells rather than biofilm cells (European Standard—EN 1276:2009). This misconception leads to the current ineffective results obtained by conventional cleaning and disinfection strategies [2].

Understanding biofilm formation might open the possibility to investigate new alternatives to control, or reduce its impact on surfaces. Biofilm formation begins when planktonic cells interact with surfaces establishing a *reversible* first adhesion [18]. At this stage, the ability of bacteria to attach to a surface is dictated by the presence of appendages and associated proteins in the surface of the cells. Once the initial electrostatic repulsion between cell and surface is overcome, the *irreversible* attachment begins [19]. This attachment is mediated by the secretion of polysaccharides and the production of adhesins [18].

All these interactions between surface and cell occur at a nanometric level. Interesting approaches have been introduced to control biofilm formation and bacterial development on surfaces by intervening at this particular nanometric scale [20]. One example of this is surface modifications with specific and highly controlled nanotextures; such as regular nanopatterns [21], which affect biofilm formation and development. However, surface patterning techniques are in their early development and very expensive.

Another nanoscale strategy consists of the use of nanomaterials and nanostructured coatings. A widely studied nanomaterial is graphene oxide (GO), which possesses a strong antimicrobial effect [22,23], due to the cell membrane disruption caused by its interaction with the functional groups present in this nanomaterial. This cytotoxic effect on bacterial cells also presents a potential risk to human health and the environment [24]. A similar effect on biofilms has also been described for silver nanomaterials and multi-walled carbon nanotubes [25,26,27]. Finally, nanoparticles of copper oxide (CuO) are usually used to reinforce antifouling paintings, in spite of its high toxicity [28] and harmful impact on marine environments and aquatic species [11].

To date, there is no known technique that successfully prevents or controls biofilms without causing adverse side effects [2]. Within this context, the search for new strategies must continue. One of the most recently developed nanomaterials is single-layer graphene, which has been poorly investigated for biofilm-control applications. Single-layer graphene (SLG) is usually produced by chemical vapor deposition, and is composed of a single-atom-thick sheet of sp^2^-bonded carbon atoms arranged in a honeycomb two-dimensional lattice [29]. Chemical vapor deposition (CVD) graphene is the most popular form of large-area graphene and reaches surface areas in the centimeters square range.

In contrast, Graphene Oxide (GO) coatings are primarily obtained by chemical oxidation of graphite [30], and it can be defined as a graphene flake with carboxylic groups at its edges, and phenol hydroxyl and epoxide groups on its basal plane [31]. This range of reactive oxygen functional groups confers antimicrobial activity and toxicity mechanisms, linked to oxidative stress [29,32]. Unlike GO coatings, SLG coatings do not possess bactericide activity, although a previous study using planktonic bacteria has shown that SLG interferes with the genetic expression of bacterial adhesion [33]. This study shows that SLG coatings considerably reduce the adhesion of bacteria in planktonic state (floating cells) and sessile state (attached cells without EPS production) due to surface interaction modification [34]. Such an initial biofilm growth stage was tuned by evaluating bacterial adhesion to SLG-coated surfaces at a short time (24 h). However, SGL coating efficiency to control the formation of biofilms at its advanced *irreversible* stage (which is, in fact, the most complex form to eradicate) has not been explored yet.

Recently, a new generation of graphene-like two-dimensional materials with an atomic structure similar to graphene, but different chemical composition and properties, have attracted widespread attention [35]. One of them, hexagonal boron nitride (h-BN), has shown similar biological performance to SLG under microbial corrosion conditions [36]. h-BN is composed of boron and nitrogen atoms in a honeycomb arrangement, consisting of sp^2^-bonded two dimensional layers [37]. There is a similarity in structure with graphene; as atoms are bound by strong covalent bonds, forming a single h-BN layer. But unlike the highly conductive graphene, h-BN possesses a wide band gap of 6 eV [38]. Such differences (and similarities) between h-BN and SGL properties could help to elucidate any connection between the interaction of graphitic-like membranes and biological systems. In particular, the lack of information regarding nanotechnological approaches to control biofilm formation at its *irreversible* stage motivated us to study the efficiency of SLG and h-BN coatings to prevent biofilm formation at such growth conditions, in contrast to planktonic or sessile state bacteria (*reversible* growth stage).

As a bacterial model, a wild strain of *Enterobacter cloacae* isolated from natural environments was selected. *Enterobacter cloacae* is a Gram-negative bacterium that belongs to the family *Enterobacteriaceae*. It has been reported that it forms biofilms in most environments, causing opportunistic infections and colonizing medical devices, being one of the ten most isolated nosocomial pathogens [10,39]. Their ability to persist in these environments, as well as their virulence, makes them a suitable model for this study. To evaluate the efficiency of SLG and h-BN coatings, we first determined the growth time at which *E. cloacae* reaches its *irreversible* biofilm stage. At that particular time the effect of those coatings on biofilm formation was studied.

## 2. Materials and Methods

### 2.1. Synthesis and Transfer

Single-layer graphene growth (for transferred samples) was performed through chemical vapor deposition (CVD) with methane as a precursor, using 25 μm thick copper foil (99.99% purity) as a synthesis substrate. The CVD growth process was performed inside a quartz tubular furnace after heating at 1000 °C under a methane-hydrogen flow rate of 20 sccm (standard cubic centimeter per minute) and 10 sccm, respectively, as reported by Parra et al. [33]. A slight modification of this methodology was introduced by supplying this mixed flux in five steps of 20 min each. Between each step, the sample was held only under the hydrogen flux for 10 min to ensure a high coverage. The final methane step was followed by rapid cooling under a hydrogen-argon flux of 10 sccm and 20 sccm, respectively. Commercial single-layer h-BN grown on Cu foil were used for this study (Graphene Supermarket, Calverton, NY, USA). The PMMA (Polymethyl methacrylate)-assisted transfer method was used in order to obtain transferred graphene and h-BN on glass [36] (See supplementary Figure S1). All graphene and h-BN samples used in this study were 1 cm^2^ in area.

### 2.2. Characterization SLG and h-BN

Scanning tunnelling microscopy (STM; UHV-VT Omicron, Uppsala, Sweden) and atomic force microscopy (AFM; Asylum Research Instruments MFP-3D, Santa Barbara, CA, USA) were used to characterize the topography of samples with nanoscale resolution. MicroRaman measurements (Renishaw, 532 nm laser, Gloucestershire, UK) were used to characterize the quality of as-grown and transferred graphene and h-BN. Contact angle measurements were performed to characterize surface hydrophobicity of coated and uncoated samples. A drop of Milli-Q water (2 μL) was placed on the surface of graphene- and h-BN-coated glass samples, and images were immediately captured using a high-resolution camera. The contact angle was measured based on image analysis [40] using the image processing software Image J with the plug-in Drop Shape Analysis (bundled with 64-bit Java 1.6.0_24, public domain) based on B-spline snakes algorithm [41].

### 2.3. Strain Isolation

*Enterobacter cloacae* strain used in this study was isolated from biofilm samples collected from aquaculture nettings off of the coast of Castro, Región de Los Lagos, Chile. This bacterial strain was isolated using the streak plate method. The sample was grown overnight on marine broth (MB) (BD Difco Marine broth 2216, NJ, USA) at 28 °C for 18 h, and spectrophotometrically standardized to reach a final absorbance of 0.1 at 600 nm (Thermo Scientific Multiskan GO, Waltham, MA, USA).

This *E. cloacae* strain was selected based upon its ability to form biofilm following the the microtiter assay described by O’Toole [42]. To identify the *E. cloacae* strain, multiple assays were conducted. Cellular morphology and biochemical tests were evaluated [43,44] (See supplementary data, Table S1). In addition, molecular identification based on the 16S rDNA sequence was accomplished using a DNA extraction and purification kit (FavorPrep^TM^ Soil DNA Isolation Mini Kit, Wembley, Australia). Gene 16S rRNA was amplified using primers F799 [45] and R1492 [46] synthesized by Integrated DNA Technologies, USA (Fermelo Biotec, Santiago, Chile). The amplified products were sequenced (Macrogen, Seoul, Korea), and analyzed using Mega6 and Basic Local Alignment Search Tool (BLAST) software (2.6.0, Rockville, MD, USA) (https://blast.ncbi.nlm.nih.gov/Blast.cgi).

### 2.4. Biofilm Formation Microtiter Assay—Biofilm Growth over Time

*E. cloacae* biofilm formation was measured using a modification of the standard method described by O’Toole [42]. Five milliliters of MB were inoculated with an isolated colony and grown overnight at 28 °C. Before use, the bacterial suspension was diluted to reach a final optical density (OD 600) of 0.1. A volume of 20 μL of the standardized inoculum was pipetted into a sterile, polystyrene 96-well flat-bottomed microtiter plate (Cell culture plate, Nest Biotech Co., Ltd., Wuxi, China) containing 180 μL of MB. A 200 μL aliquot of the diluted bacterial suspension was added to each growth control well. The negative control wells contained 200 μL of broth medium only. The plate was incubated aerobically at 28 °C for different periods of time (24, 48, 72, and 96 h). The culture medium was refreshed in one set of samples every 24 h. After incubation, the culture media was carefully removed from the wells and washed three times with 200 μL of phosphate-buffered saline (PBS) to remove the non-adherent bacteria.

The plates were then left to air dry under sterile laminar flow in a safety biosecurity cabinet (Nuaire NU-425 Class II, Type A2, Plymouth, MN, USA) for 1 h. Cells adhered to the plate were stained with 200 μL of 0.1% (*w*/*v*) crystal violet (Merck, Damm, Germany) for 30 min. The plates were carefully rinsed off under running tap water to remove excess stain, and air-dried under sterile laminar flow at room temperature. Bound dye was dissolved with 200 µL of 95% (*v*/*v*) ethanol. The optical density (OD) of each well was measured at 590 nm using a microtiter plate reader (Thermo Scientific Multiskan^TM^ GO Microplate Spectrophotometer), using ethanol 95% as blank. To quantify the biofilm formed on each experiment, six replica wells were used per experiment and three independent experiments were performed.

### 2.5. Colored Staining in Bright Light Microscopy—Effect of Media Replacement on Biofilm Growth

A 100 µL inoculum of *E. cloacae* strain suspension (OD_600nm_ at 0.1) was transferred to a glass coverslip (18 mm × 18 mm, Sail Brand, Haimen, China) and incubated in a sterile petri dish at 25 °C for different periods of time (24, 48, 72, and 96 h). Two different sets of samples were carried out separately; one with media replacement every 24 h, and the other set with no media replacement. After the corresponding incubation time, safranin staining, Alcian blue/safranin staining, and Gram staining were performed on the samples, separately. The samples were observed by bright-field microscopy on the 100× objective lens (Carl Zeiss Axiostar Plus Transmitted-Light Microscope, Oerzen, Germany).

### 2.6. Inhibition of Biofilm Growth in Coated Surfaces

A 100 µL inoculum of standardized *E. cloacae* bacterial suspension was transferred to a 1 × 1 cm^2^ coated glass samples (microscope slides Cat No. 7105, Sail Brand, China); graphene-coated glass, h-BN-coated glass, and uncoated glass (triplicate test for all samples). Samples were incubated in a sterile petri dish at 28 °C for 48 h. Marine broth media was replaced on all samples at 24 h. Once the incubation period had elapsed, media was removed, and samples were washed in sequence with sterile water, phosphate buffered saline (PBS), and sterile water. Once the samples were completely dried under sterile laminar flow, 100 µL of 0.1% crystal violet solution was pipetted on the surface and samples were incubated for 30 min at room temperature. Unbound dye was removed by several rinses with sterile water until no more dye was solubilized. Samples were air dried under sterile laminar flow. Once completely dry, samples were transferred to a 6-well cell culture plate (TrueLine cell culture plate TR5000 6 well, polystyrene, sterile, non-pyrogenic, San Jose, CA, USA), and adhered dye was solubilized with 100 µL of ethanol 95% (Merck, Germany). The rinsed solution was pipetted onto a sterile 96-well (Cell culture plate, Nest Biotech Co., Ltd., China). The optical density (OD) of each well was measured at 590 nm using a microtiter plate reader (Thermo Scientific Multiskan^TM^ GO Microplate Spectrophotometer), using 95% ethanol as blank. To quantify the biofilm formed on each experiment, six replica wells were used per experiment and three independent experiments were performed.

### 2.7. Scanning Electron Microscopy Images

Scanning electron microscopy (SEM) (Carl Zeiss, EVO MA-10) was used to visually evaluate the architecture of biofilms and coated surfaces with microscale resolution. A 100 µL inoculum of standardized *E. cloacae* bacterial suspension was transferred to graphene-coated glass, h-BN-coated glass, and uncoated glass (triplicate evaluation for all samples). Samples were incubated in a sterile petri dish (90 mm × 15 mm, Cat. No. 752001, Nest Biotech Co., Ltd., China) at 28 °C for 48 h. Growth media was replaced on all samples at 24 h. Once the incubation period had elapsed, media was removed, and samples were washed sequentially with sterile water, PBS, and sterile water. Samples were air-dried under sterile laminar flow. Samples were fixed with 3.0% (*w*/*v*) glutaraldehyde solution for 24 h. The samples were dehydrated by washing with a graded ethanol series (from 10% to 100%) for 3 min each, followed by critical-point drying and gold coating. Scanning Electron Microscopy (SEM) images were recorded using a Carl Zeiss microscope (EVO MA-10).

### 2.8. Epifluorescence Essay

An inoculum of *E. cloacae* bacterial suspension was transferred onto graphene-coated glass, h-BN-coated glass, and uncoated glass samples (triplicate evaluation for all samples), and incubated following the procedure described in Section 2.6. After the incubation media was removed and samples were washed with sterile water and PBS, and dried under sterile laminar flow. Epifluorescence assays were performed using the L7012 Live/Dead backlight bacterial viability kit protocol. Samples were submerged into solutions provided in the same kit (0.01 mM of Syto9 and 0.06 mM of propidium iodide). Samples were kept in the dark for 15 min and then observed under a fluorescent optical microscopy (Carl Zeiss Axiolab A1microscope).

### 2.9. Viability of Planktonic Cells Assay—State of Non-Attached Bacteria

After 48 h incubation of *E. cloacae* on coated samples, the viability of non-adhered bacteria was quantified. This allows the evaluation of any possible bactericide activity of graphene and h-BN coatings over bacteria in planktonic state (bacteria that did not reach its biofilm *irreversible* growth stage). *E. cloacae* was incubated for 48 h as described in previous sections. After that period, bacteria non-attached to samples (bacteria suspended in media) were recovered using a standard micropipette. Cell viability was determined using the microdot methodology in a trypticase soy agar (TSA) plate [33]. A volume of 20 μL of the bacteria recovered from each sample surface was pipetted into a sterile, polystyrene 96-well flat-bottomed microtiter plate (Cell culture plate, Nest Biotech Co., Ltd., China), containing 180 μL of tryptic soy broth (TSB) and then diluted each sample until obtaining a dilution of 1 × 10^−8^. Five µl aliquots of every dilution were inoculated in TSA plates to obtain CFU/mL. Each experimental trial was conducted in triplicate. 

## 3. Results and Discussion

### 3.1. E. cloacae Biofilm Growth

To evaluate the efficiency of the nanometric coatings on biofilm adhesion, it is necessary to first evaluate the integrity of the biofilm over time. In fact, most studies focus on bacterial adhesion during the first 24 h of contact between the bacteria and surface [47,48,49], without evaluating if the biofilm has reached its *irreversible* stage (with the presence of EPS) or not. The efficiency of our nanostructured coatings for reduction of biofilm growth required testing under typical conditions for *irreversible* biofilm growth and before biofilm detachment (Figure S2; for details of biofilm growth cycle).

**Figure 1:**
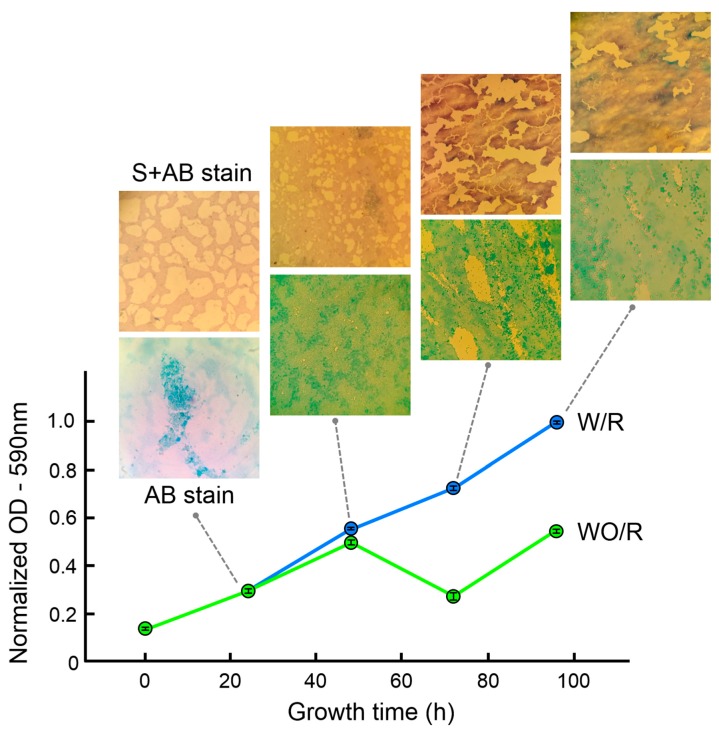
Optical density (OD) measurements and optical micrographs of a staining test of *E. cloacae* biofilms grown on glass as a function of time. The response of biofilm under no-media replacement incubation conditions (WO/R) and media replacement incubation conditions (W/R) is depicted. Corresponding staining test for each time is included (for optical micrographs of staining test for WO/R conditions see supplementary Figure S3).

Biofilm growth curve as a function of time can be obtained by means of optical thickness (optical density—OD), measured as intensity reduction of a light beam transmitted through the biofilm, which correlates with biofilm mass. The kinetic growth curves shown in the plot of Figure 1, quantify the adhered biomass on a glass surface over a 96-h period. 

Characteristics of the growth media used for bacterial incubation can affect biofilm growth [49]. Considering our experimental conditions; where nutrients in the incubation medium were constantly consumed by bacteria, we explored the effect of biofilm replacement on biofilm growth every 24 h.

The curve in green illustrates the behavior of a biofilm under starvation (no media replacement incubation–WO/R). Simultaneously, the curve in blue depicts the response under media replacement conditions incubation (W/R). Both curves showed similar results during the first 48 h of incubation regardless of the media conditions applied. After this 48-h period of incubation, the curves showed different patterns of behavior. The media replacement condition showed a consistent increment of growth from 48 to 96 h. The no-media replacement condition presented a decrease in growth, which was later reversed at time 96 h.

The difference in the curves’ behavior may be explained by the different stages of biofilm development, where stages of detachment may take place after reaching a critical biofilm size. To support this interpretation, we carried out staining tests on the samples at the same growth time as OD analysis. To visualize the architecture of the biofilm, a safranine/Alcian blue staining was used. The presence of exopolysaccharides in the samples was identified using Alcian blue [50]. This staining method is specific for polysaccharide components, and in this case, can be applied to identify the biofilm matrix. Safranine stains bacterial cells in contrast to Alcian blue, which mainly stains EPS [51].

Evaluation of biofilm growth using safranine (S) and Alcian blue (AB) stains (optical microscope images in Figure 1) was in agreement with OD results, showing at 72 h the breakdown of biofilm architecture (EPS and bacteria), presumably connected to biofilm removal by detachment. At 48 h completeness of biofilm was observed using both staining methods, indicating this is the time in the *irreversible* growth stage of biofilm where the maximum biomass size adhered to the surface.

### 3.2. Morphology of E. cloacae Biofilm at Reversible and Irreversible Stages

**Figure 2:**
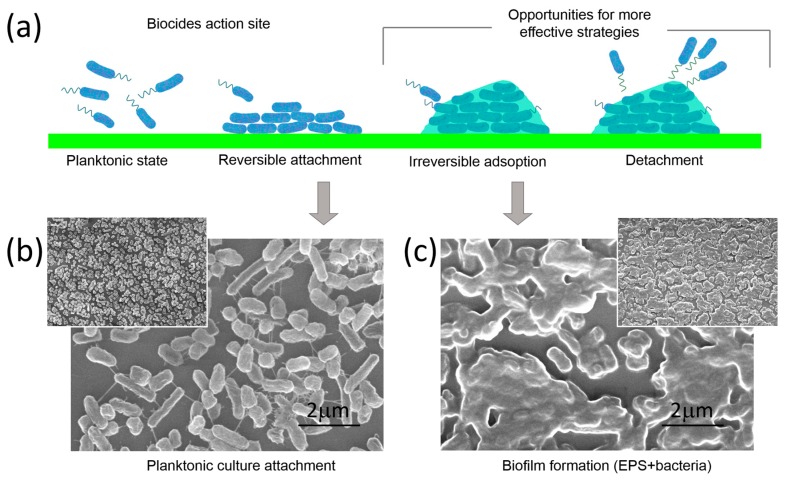
*Enterobacter cloacae* biofilm formation on glass. (**a**) Illustrative diagram of biofilm formation stages: Planktonic state, *reversible* attachment, *irreversible* adsorption, and detachment; (**b**) Scanning electron image (SEM) of *E. cloacae* after 24 h incubation on a glass surface. *Reversible* attachment can be observed by the presence of bacterial surface filaments; and (**c**) SEM image of *E. cloacae* after 48 h incubation on a glass surface. Biofilm formation can be seen by the presence of exopolymeric substances. For more SEM images of biofilm formation stages see Figure S2.

To extend the discussion regarding the stage of *E. cloacae* biofilm formation, Figure 2 presents SEM micrographs of glass samples exposed to *E. cloacae* before and at the obtained optimal biofilm growth time. 

As it was previously discussed, biofilm formation is a complex process, and bacteria must undergo a series of different stages to transform from a planktonic state to *irreversible* adsorption (Figure 2a). Planktonic bacteria following Brownian motion will verge to the surface led by long-range forces [52], the presence of fimbriae and flagella appendages will disrupt the initial electrostatic repulsion between the cell and substratum, and the *reversible* attachment stage will begin. During this phase, bacteria still show Brownian motion and can be easily removed by cleaning [18].

SEM micrograph of the *reversible* attachment stage in the *E. cloacae* biofilm growth (24 h incubation) is shown in Figure 2a. Aggregation and cohesion of bacteria occur, and the presence of bacterial cell appendages (see filaments in SEM image) assists in the attachment of bacteria to the surface or to each other. When these bacterial cells start their transition from this *reversible* to the *irreversible* stage, bacterial cell filaments retract and adhesins mediate attachment by a more intimate contact between bacteria and surface [18]. The *irreversible* attachment finishes when bacteria consolidate the adhesion process by secreting exopolymeric substances. This adhesion to the surface becomes *irreversible*, and bacteria cannot be removed by gentle rinsing [19]. This is clearly observed in Figure 2c (48 h incubation), where the presence of EPS is the critical difference between *reversible* and *irreversible* attachment. In fact, individual bacteria embedded into the EPS wrapping can be distinguished in the SEM micrograph. This allows identifying the same bacterial concentration (density) over the glass surface in both, the *reversible* (Figure 2b) and *irreversible* stage (Figure 2c).

This qualitative microscopy analysis again confirmed data presented in Figure 1, which indicated that a growth time of 24 h led to an under-incubated biofilm for adhesion experiments. These results indicated that a 48-h incubation time has to be chosen when working with *E. cloacae* in order to be in its *irreversible* adhesion regime but without presenting detachment for over-incubation. Any assays to demonstrate the effect on biofilm formation of coated surfaces must consider this for the design of the experimental conditions.

### 3.3. Characterization of Nanostructured Coated Samples

**Figure 3:**
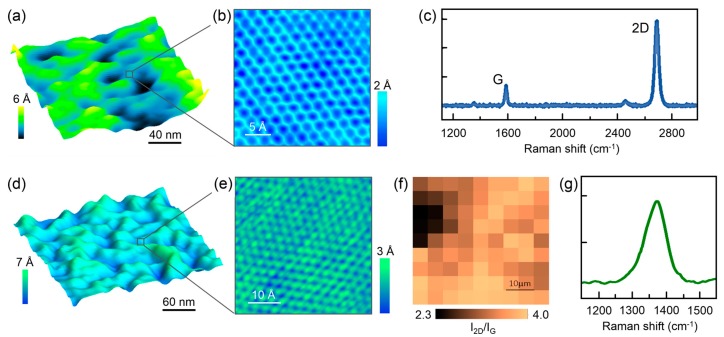
Characterization of h-BN and graphene coatings. (**a**) Atomic force microscopy image of SLG transferred onto glass; (**b**) Scanning tunneling microscopy atomic-resolved image of SLG; (**c**) representative Raman spectrum of SLG; (**d**) STM image of h-BN; (**e**) STM atomic-resolved image of h-BN; (**f**) Raman map of SLG; and (**g**) representative Raman spectrum of h-BN.

Characterization of as-grown h-BN and SLG on Cu, h-BN, and SLG samples transferred onto glass was performed (Figure 3). The terms graphene or SLG will be used interchangeably. The microstructure was evaluated using scanning electron microscopy (SEM) and atomic force microscopy (AFM) whereas topography (with atomic and nanometric resolution), graphitic quality and composition were evaluated through a combination of scanning tunneling microscopy (STM) and Raman spectroscopy.

Cells of most strains of bacteria are typically 1 micrometer in diameter. Surface roughness on the μm or larger scale and topography irregularities serve as ‘hideouts’ from unfavorable environments. At the same time, they provide a larger surface area, hence higher hosting capacity, and enhanced bacteria-substrate interaction [53]. In our case, surface roughness obtained from STM images was less than 1 nm (~0.3 nm) for both graphene- and h-BN-coated samples (see Supplementary Figure S4). Surface roughness at the nanometer scale has been shown to increase adhesion [54], but differences in surface roughness on the order of tens of nanometers has been found to be negligible in comparison to other surface properties such as charge [55].

AFM images of single-layer graphene (SLG) grown on Cu, and transferred onto glass showed less than 1 nm corrugation, which is presumably connected to a strain relaxation mechanism generated during the transfer process (Figure 3a). Similar features were found in h-BN transferred onto glass (Figure 3d). Atomic-resolved images of SLG and h-BN on glass obtained using STM (Figure 3b,e) exhibit the distinctive honeycomb structure with an in-plane lattice parameter of 2.5 Å (for SLG) and 2.4 Å (for h-BN), respectively, consistent with literature values [56,57]. Few signs of surface contamination were found by this atomic-resolved technique. 

To verify the graphitic quality of graphene coatings micro-Raman spectroscopy measurements were performed. SLG typically display sharp G (1584 cm^−1^) and 2D (2680–2693 cm^−1^) bands (Figure 3c). Micro-Raman spectroscopy mapping was carried out on a 50 μm × 50 μm area of graphene transferred onto glass in order to obtain spatial-resolved information regarding ratio of the intensities I_2D_ and I_G_ of the bands 2D and G (I_2D_/I_G_) (Figure 3f). It is known that the ratio I_2D_/I_G_ is dependent on the number of graphene layers. The ratio I_2D_/I_G_ >2 is for monolayer graphene, 2 > I_2D_/I_G_ > 1 for bilayer graphene, and I_2D_/I_G_ <1 for multilayer graphene [58,59]. SLG transferred onto glass samples always exhibits a I_2D_/I_G_ ratio larger than 2, confirming the graphitic quality of the samples. Multiple areas of h-BN samples were analyzed and the representative spectrum is shown in Figure 3g. These results are consistent with single layer h-BN, according to values reported in the literature [60].

### 3.4. Biofilm Formation on h-BN- and Graphene-Coated Samples

**Figure 4:**
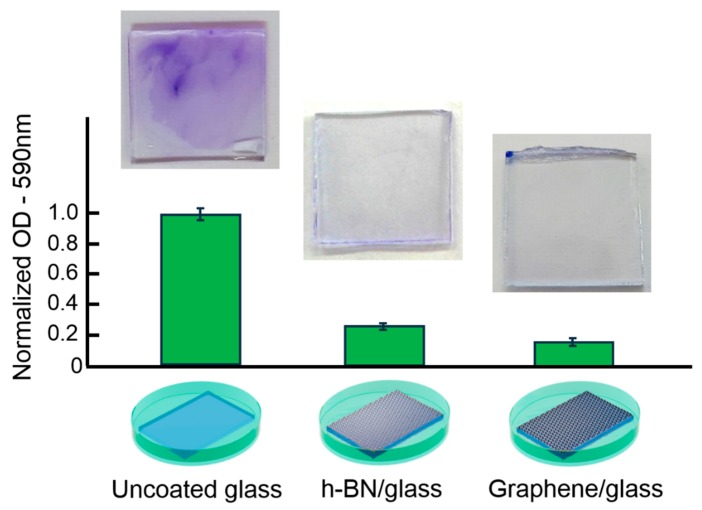
Optical density (OD) and crystal violet results for *E. cloacae* biofilms grown on uncoated glass, h-BN-coated glass, and SLG-coated glass samples.

Gram staining tests were performed as a first approach to characterize biofilm formation on coated and uncoated glass samples (Figure 4). A well-known method to quantify adhered biomass is the microtiter assay, which uses crystal violet staining. This test allows an indirect quantification of the adhered biomass, as the optical density of bacterial biofilms stained with this dye indicates the concentration of bacteria, and is used as an index of adherence [42].

Figure 4 shows results of optical density for the *E. cloacae* biofilm formed at nanoscale-modified samples after 48 h of exposure. This quantification of biomass formation revealed that graphene-coated glass exhibits 83.6% less biofilm than uncoated glass. In the case of h-BN, a 73.8% suppression of biofilm formation was found. Crystal violet staining results for each sample are also shown in Figure 4. A darker blue color on the uncoated glass surface indicates the effect of the dye staining different layers of the biofilm. Coated glass surfaces showed no apparent coloration under this dye technique, in agreement with the OD results.

Although crystal violet staining quantified the adhered biofilm, it did not give information regarding the state of the cells in the biofilm, as it cannot differentiate between live or dead bacteria.

**Figure 5:**
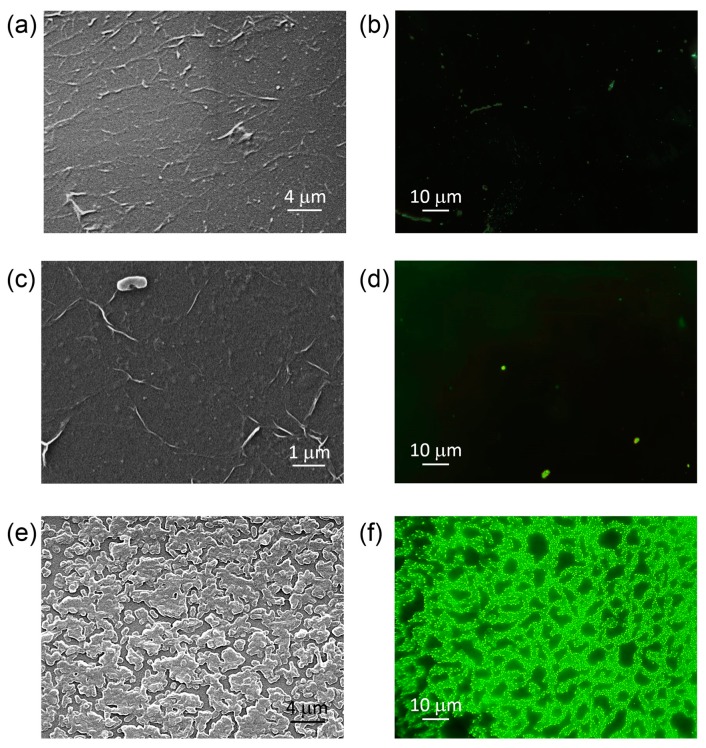
Scanning electron microscopy and epifluorescence images of *Enterobacter cloacae* biofilms’ distribution on coated and uncoated glass samples. SEM images of samples after *E. cloacae* 48 h incubation: (**a**) Graphene-coated glass, (**c**) h-BN-coated glass, and (**e**) uncoated glass. Corresponding epifluorescence microscopy micrographs for (**b**) graphene-coated glass, (**d**) h-BN-coated glass, and (**f**) uncoated glass.

In order to characterize bacterial adhesion to graphene-coated, h-BN-coated, and uncoated glass samples, SEM and fluorescence microscopy analyses were carried out. Morphology of *E. cloacae* incubated for 48 h on graphene-coated glass, h-BN-coated glass, and uncoated glass samples are shown in Figure 5a,c,e, respectively.

SEM images of graphene and h-BN transferred onto glass after 48 h incubation show the absence of attached bacteria or biofilm formation (Figure 5a,c, respectively).

Wrinkles can be identified across the coated samples surface. These structures are usually considered to be a result of compressive stress during cooling caused by the difference in thermal expansion coefficient between two-dimensional layered materials and substrates [38].

After 48 h incubation, the uncoated glass surface was fully covered by *E. cloacae* biofilm, which extended like a percolated network (Figure 5f). Although bacteria were embedded within its extracellular polymeric substances, they still could be individually distinguished (see Figure 2 for comparison).

In order to identify the state of bacteria in such a biofilm structure, epifluorescence microscopy analysis was performed. When using a dead-live kit, green staining is an indication of live bacteria, whereas, red stained bacterial bodies are indicative of dead bacteria. Representative epifluorescence microscopy images of graphene-coated glass, h-BN-coated glass, and uncoated glass samples after 48 h incubation are shown in Figure 5b,d,f, respectively.

Fluorescence microscopy results for uncoated glass samples (Figure 5f) showed that all bacteria present in *E. cloacae* biofilm were in fact alive. In Figure 5b,d, the absence of substantial bacterial adhesion on the graphene-coated and h-BN-coated surfaces concurred with the analyses of OD measured to quantify biofilm formation on these samples. The scarcity in adherence could be related to the inability of these microorganisms to form biofilm (bacteria + EPS) in the presence of these nanostructure-coated surfaces. The lower OD observed in the quantification of biofilm formation and the results obtained by fluorescence microscopy may suggest that bacteria remain in planktonic state, as seen in Figure 2. Fluorescence results for h-BN-coated and graphene-coated glass samples after 48 h of *E. cloacae* incubation were in agreement to SEM images, in terms of the absence of attached bacteria or biofilm. It is important to mention that samples for SEM imaging were prepared through critical-point drying, which applies considerable force on the specimen (present at the phase boundary as the liquid evaporates). This can cause biofilms to detach from the glass surface. Samples for fluorescence imaging, in contrast, do not undergo this aggressive treatment, as it is not needed for this analysis. The absence of bacteria in fluorescence images of coated samples confirms the validity of our SEM results.

Epifluorescence and SEM results suggested both nanostructured coatings suppress dramatically bacterial attachment, which is determinant to biofilm and fouling formation. 

To evaluate the state of microorganisms that did not adhere to the nanoscale-modified samples after 48 h, we performed viability tests on the recovered planktonic state bacteria. Recent reports have connected the high-conductance properties of graphene coatings with antibacterial activity [61]. Such effect is claimed to be connected to an increase in the electron exchange between bacteria and graphene. According to our viability results (Figure S5), no bactericide effect was found; neither in recovered planktonic state bacteria (non-adhered bacteria) after exposition to graphene (conducting), nor after exposure to h-BN (insulating). This suggested that electrical conductivity of these nanostructured coatings does not induce bactericide activity. Our results also indicated that coating conductivity is not related to the observed inhibition of biofilm formation. 

Unlike other nanomaterials such as graphene oxide, which suppress biofilm formation by membrane rupture leading to cell death [23], our viability results indicated that graphene and h-BN coatings have a different inhibition mechanism of biofilm formation, probably related to long-range and medium-range interactions. In the next section, we explore the influence of electrostatic forces and surface energy on bacterial adhesion on coated surfaces as a possible source of the observed inhibition of biofilm formation.

### 3.5. Nanostructured Coating Effects on Surface Energy and Electrostatic Interaction

As soon as microorganisms reach a surface, they will be attracted or repelled by it, depending on the sum of the different non-specific interactions [62,63]. The first relevant interaction in this system is the one related to long-range electrostatic forces between graphene-coated glass surfaces and cells that might be affecting the initial (and *reversible*) bacterial adhesion process. Charge contributes to bacterial adhesion to either living or inanimate surfaces. It has been suggested that bacteria, when introduced into aqueous suspensions, are always negatively charged [64]. To determine if electrostatic long-range interactions between graphene-coated glass and bacteria contribute to an initial repulsion between bacteria and the graphene-coated substrate, we performed theoretical calculations to obtain electrostatic force *F*(*r*) between bacteria and material surface (glass, graphene-coated glass, and h-BN-coated glass) as a function of their separation distance using the expression [65]:

$$F\left(r\right)=\frac{2\pi d_{1}d_{2}\varepsilon \varepsilon_{0}\kappa \;}{d_{1}+d_{2}}{\left(\frac{k_{B}T}{ze}\right)}^{2}\frac{\phi_{1}^{2}+\phi_{2}^{2}+\left(2e^{r\kappa }\phi_{1}\phi_{2}\right)}{\left(e^{r\kappa }+1\right)\left(e^{r\kappa }-1\right)}$$

where F is electrostatic force (in N); r is distance between bacteria and surface (in m); d is the radius of bacteria (or glass piece, or graphene-coated glass, or h-BN-coated glass; in m); ε is the dielectric constant of water [66] (78.43 at 25 °C); $\varepsilon_{0}$ is the permittivity of free space (8.854 × 10^−12^ C/Jm); $k_{B}$ is Boltzmann’s constant (1.381 × 10^−23^ J/K); T is temperature (25 °C); z is the valence of electrolyte ions (1 for NaCl); and e is the charge of an electron (1.602 × 10^19^ C). The inverse Debye length κ describes the thickness of the electrostatic double layer of counter-ions that surrounds charged parts of the system (bacteria or glass) in solution. For monovalent electrolytes (e.g., NaCl), $\kappa^{-1}$ is given by $0.304/{\left(c\right)}^{1/2}$ (in 1/nm), where c is the concentration of the electrolyte (in mol/L) and contains information of ionic strength of solution [67]. In our case, we evaluate 2% (*w*/*v*), concentration of suspension media (marine broth medium). Surface potential ϕ is described by $ze\psi /k_{B}T$, where ψ is the surface potential of the bacteria, glass piece, graphene-coated glass piece, and h-BN-coated glass piece (in V). The values of the surface potentials of glass piece, graphene-coated glass piece, h-BN-coated glass piece and *Enterobacter* are −55 mV [68], −77 mV [69], −50 mV [70] and −27 mV [71], respectively. 

**Figure 6:**
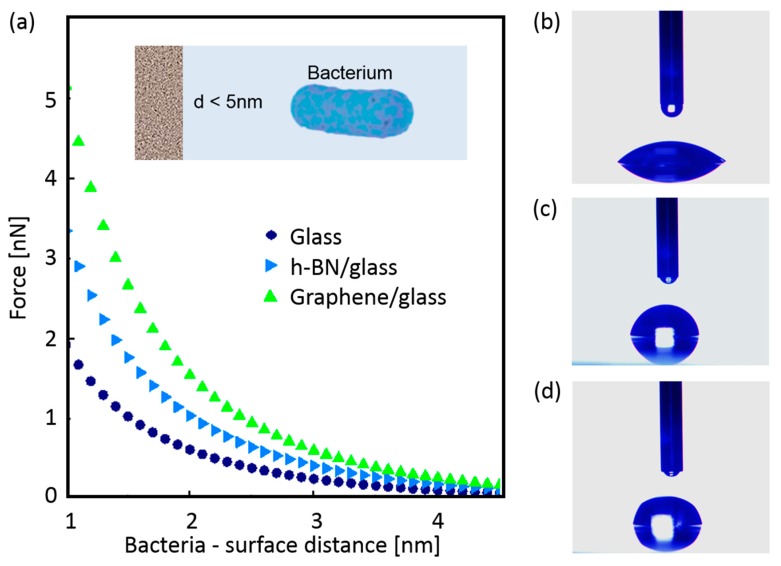
(**a**) Theoretical calculations of electrostatic force as a function of the distance between bacteria and surface (uncoated and coated glass samples). Images of contact angle measurements for (**b**) glass, (**c**) h-BN-coated glass, and (**d**) graphene-coated glass samples.

Figure 6a shows the theoretical force-distance relationship for the bacteria-surface system. According to this result, the electrostatic force in this system is expected to be a repulsive and short range (<5 nm). Although electrostatic repulsion between bacteria and glass always increases when the glass is coated with h-BN or graphene, this effect is stronger for graphene-coated surfaces when a bacterium cell approaches the surface.

An even shorter-range interaction of hydrophobic nature occurs when the bacteria-surface distance is smaller than 1.5 nm (if bacteria are capable of overcoming this initial electrostatic repulsion). Such hydrophobic characteristics are mainly determined by physicochemical surface properties and influence the attachment of bacteria to surfaces [72]. We performed contact angle measurements in order to determine the influence of possible hydrophobic or hydrophilic characteristics of the nanostructured coatings over bacterial adhesion. Figure 6 shows the results for glass (Figure 6b), graphene-coated glass (Figure 6c), and h-BN-coated glass samples (Figure 6d). According to our measurements, a transition from a strongly hydrophilic surface (contact angle of ~42.4° ± 0.7°) for glass substrate to light hydrophilic surface for h-BN-coated glass (contact angle of ~76.8° ± 0.5°), and graphene-coated glass (contact angle of ~81.6° ± 0.3°) was found. 

Contact angles are related to the surface free energies [73]. When the contact angle increases (for coated samples), surface wettability decreases (lower surface energy) making it more difficult for the biofilm to spread across the material’s surface [74]. These results show that both interactions, electrostatic (<5 nm range) and hydrophobic-hydrophilic (<1.5 nm range), are presumably affecting the bacterial attachment process.

## 4. Conclusions

We present a study on the performance of graphene and hexagonal boron nitride coatings to reduce *E. cloacae* biofilm formation. In particular, we focused on evaluating their efficiency at the *irreversible* stage of formation (in contraposition to planktonic state), where most of biocide solutions have a poor performance.

According to our experimental and theoretical results, graphene and h-BN coatings modify surface energy and electrostatic interactions with a biological system, which determines a significant reduction in biofilm formation. The fact that the inhibition of biofilms formation is found in both nanostructured coatings (a conducting graphene and an isolating h-BN) indicates electron exchange is not related to this effect.

In addition, no bactericidal effects were found after interaction of the biological system with graphene and h-BN coatings, indicating that the mechanism involved in the inhibition of biofilm formation of such surfaces is not biocidal in nature, like the one used for commercial products designed for this application.

These nanostructured coatings offer an environmentally friendly solution for biofilm and fouling control in a wide range of applications such as the one related to biomedical and industrial sectors. Although international standards regarding biofilm control and prevention are focused on attacking planktonic cells rather than biofilm cells, which is highly ineffective, in this work we provide a successful strategy to reduce biofilm formation which is alternative to biocides and other chemical solutions.

Future work should be focused on evaluating the efficiency of using natural biofilms that are more complex due to their heterogeneous nature.

## Supplementary Materials

The following are available online at http://www.mdpi.com/2079-4991/9/1/49/s1, Figure S1: Polymethyl methacrylate (PMMA)-assisted transfer method; Figure S2: Scanning Electron Microscopy mages of biofilm growth stages; Figure S3: Optical density (OD) results for biofilms grown with and without media replacement; Figure S4: Roughness analysis of coated glass samples; Figure S5: Cell viability of *E. cloacae* in planktonic state; Table S1: Biochemical test for bacterial strain identification.
