# Addendum: Zurob, E., et al. Inhibition of Wild *Enterobacter cloacae* Biofilm Formation by Nanostructured Graphene- and Hexagonal Boron Nitride-Coated Surfaces. *Nanomaterials* 2019, *9*, 49

**DOI:** 10.3390/nano10010138

**Published:** 2020-01-12

**Authors:** Elsie Zurob, Geraldine Dennett, Dana Gentil, Francisco Montero-Silva, Ulrike Gerber, Pamela Naulín, Andrea Gómez, Raúl Fuentes, Sheila Lascano, Thiago Henrique Rodrigues da Cunha, Cristian Ramírez, Ricardo Henríquez, Valeria Del Campo, Nelson Barrera, Marcela Wilkens, Carolina Parra

**Affiliations:** 1 Laboratorio Nanobiomateriales, Departamento de Física, Universidad Técnica Federico Santa María, Avenida España 1680, Valparaíso, Chile; 2 Laboratorio de Microbiología Básica y Aplicada, Universidad de Santiago de Chile, Avenida Libertador Bernardo O’Higgins 3363, Santiago, Chile; 3 Faculty Environment and Natural Science, Institute of Biotechnology, Brandenburg University of Technology, Universitätsplatz 1, 01968 Senftenberg, Germany; 4 Facultad de Ciencias Biológicas, Pontificia Universidad Católica de Chile, Alameda 340, Santiago, Chile; 5 Departamento de Industrias, Universidad Técnica Federico Santa María, Avenida España 1680, Valparaíso, Chile; 6 Departamento de Mecánica, Universidad Técnica Federico Santa María, Avenida Vicuña Mackenna 3939, Santiago, Chile; 7 Departamento de Física, CTNanotubos, Universidade Federal de Minas Gerais, Belo Horizonte 31310260, Brazil; 8 Departamento de Ingeniería Química y Ambiental, Universidad Técnica Federico Santa María, Avenida España 1680, Valparaíso, Chile; 9 Departamento de Física, Universidad Técnica Federico Santa María, Avenida España 1680, Valparaíso, Chile

The authors wish to make the following corrections to this paper [1]:

The acknowledgement section needs to be corrected. The updated information is included below:

**Funding:** This work was financially supported by Fondecyt Regular 1180702, FONDEF ID15I10576, Proyecto Interno Multidisciplinario PIM_USM_12_18, Fondecyt Postdoctoral N°3160568, and Millennium Science Initiative P10-035F.

This addendum does not cause any changes to the results or conclusions in the original published paper.

The authors would like to apologize for any inconvenience caused to the readers by these changes.
