# Supplementary material for: Inhibition of Wild Enterobacter cloacae Biofilm Formation by Nanostructured Graphene- and Hexagonal Boron Nitride-Coated Surfaces
Source: Nanomaterials (Basel). 2019 Jan 2;9(1):49. doi: 10.3390/nano9010049 (PMC6358881; doi:10.3390/nano9010049)
Supplement: Supplementary file 1 [file nanomaterials-09-00049-s001.pdf]

Supplementary data

Inhibition of Wild *Enterobacter cloacae* Biofilm Formation by Nanostructured Graphene- and Hexagonal Boron Nitride-Coated Surfaces

Elsie Zurob [1,2], Geraldine Dennett [1], Dana Gentil [1], Francisco Montero-Silva [1], Ulrike Gerber [3], Pamela Naulín [4], Andrea Gómez [4], Raúl Fuentes [5], Sheila Lascano [6], Thiago Henrique Rodrigues da Cunha [7], Cristian Ramírez [8], Ricardo Henríquez [9], Valeria del Campo [9], Nelson Barrera [4], Marcela Wilkens [2] and Carolina Parra [1,*]

[1] Laboratorio Nanobiomateriales, Departamento de Física, Universidad Técnica Federico Santa María, Avenida España 1680, Valparaíso, Chile; elsie.zurob@usach.cl (E.Z.); g.dennett@gmail.com (G.D.); dana.gentil@usm.cl (D.G.); monteroster@gmail.com (F.M.-S.)

[2] Laboratorio de Microbiología Básica y Aplicada, Universidad de Santiago de Chile, Avenida Libertador Bernardo O'Higgins 3363, Santiago, Chile; marcela.wilkens@usach.cl

[3] Faculty Environment and Natural Science, Institute of Biotechnology, Brandenburg University of Technology, Universitätsplatz 1, 01968 Senftenberg, Germany; gerberu@b-tu.de

[4] Facultad de Ciencias Biológicas, Pontificia Universidad Católica de Chile, Alameda 340, Santiago, Chile; pnaulin@uc.cl (P.N.); agomez@bio.puc.cl (A.G.); nbarrera@bio.puc.cl (N.B.)

[5] Departamento de Industrias, Universidad Técnica Federico Santa María, Avenida España 1680, Valparaíso, Chile; raul.fuentes@usm.cl

[6] Departamento de Mecánica, Universidad Técnica Federico Santa María, Avda. Vicuña Mackenna 3939, Santiago, Chile; sheila.lascano@usm.cl

[7] Departamento de Física, CTNanotubos, Universidade Federal de Minas Gerais, Belo Horizonte 31310260, Brazil; thiago.cunha@ctnano.com.br

[8] Departamento de Ingeniería Química y Ambiental, Universidad Técnica Federico Santa María, Avenida España 1680, Valparaíso, Chile; cristian.ramirez@usm.cl

[9] Departamento de Física, Universidad Técnica Federico Santa María, Avenida España 1680, Valparaíso, Chile; ricardo.henriquez@usm.cl (R.H.); valeria.delcampo@usm.cl (V.d.C.)

* Correspondence: carolina.parra@usm.cl

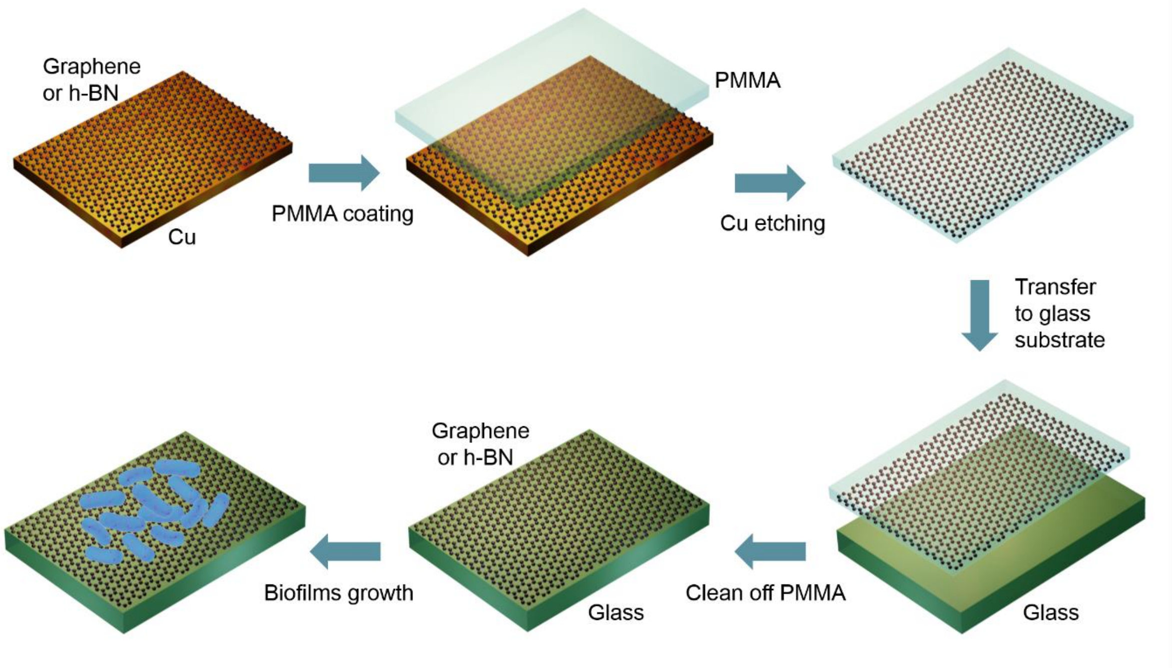

Figure S1. Illustrative diagram showing the PMMA-assisted transfer method used to obtain graphene- and h-BN-coated glass samples for the present study. A thin layer of PMMA (Polymethyl methacrylate) on graphene, on Cu (and h-BN on Cu) foil was produced by spin coating. The polymer provides a supportive framework for the two-dimensional (2D) coating before the transfer. The Cu substrate underneath was then etched away using an ammonium persulfate ((NH4)2SO8) solution. After the Cu foil was completely dissolved, the floating membrane was scooped and placed onto glass. After drying, the polymeric film was dissolved with acetone.

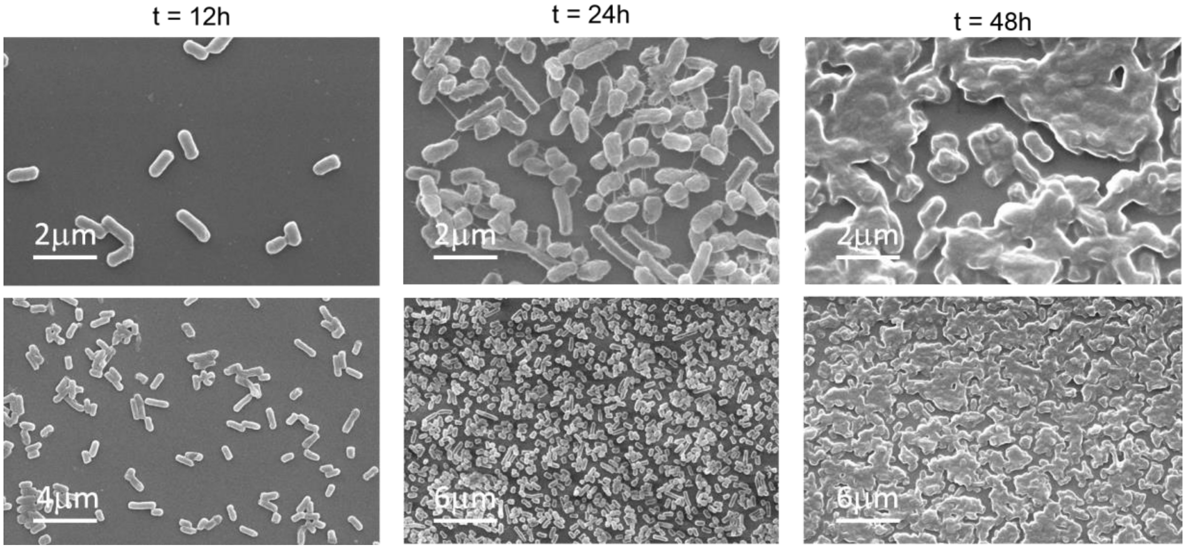

Figure S2. SEM image of *E. cloacae* after 12 h, 24h and 48h incubation on a glass surface. Reversible stage of biofilm formation can be observed by, first, the presence of bacteria adhered to the surface (12h) and then the production of filaments that assist a robust bacterial adhesion (24h). After 48 h incubation on a glass surface, the irreversible stage of biofilm growth is reached with bacteria encapsulated in exopolymeric substances (EPS).

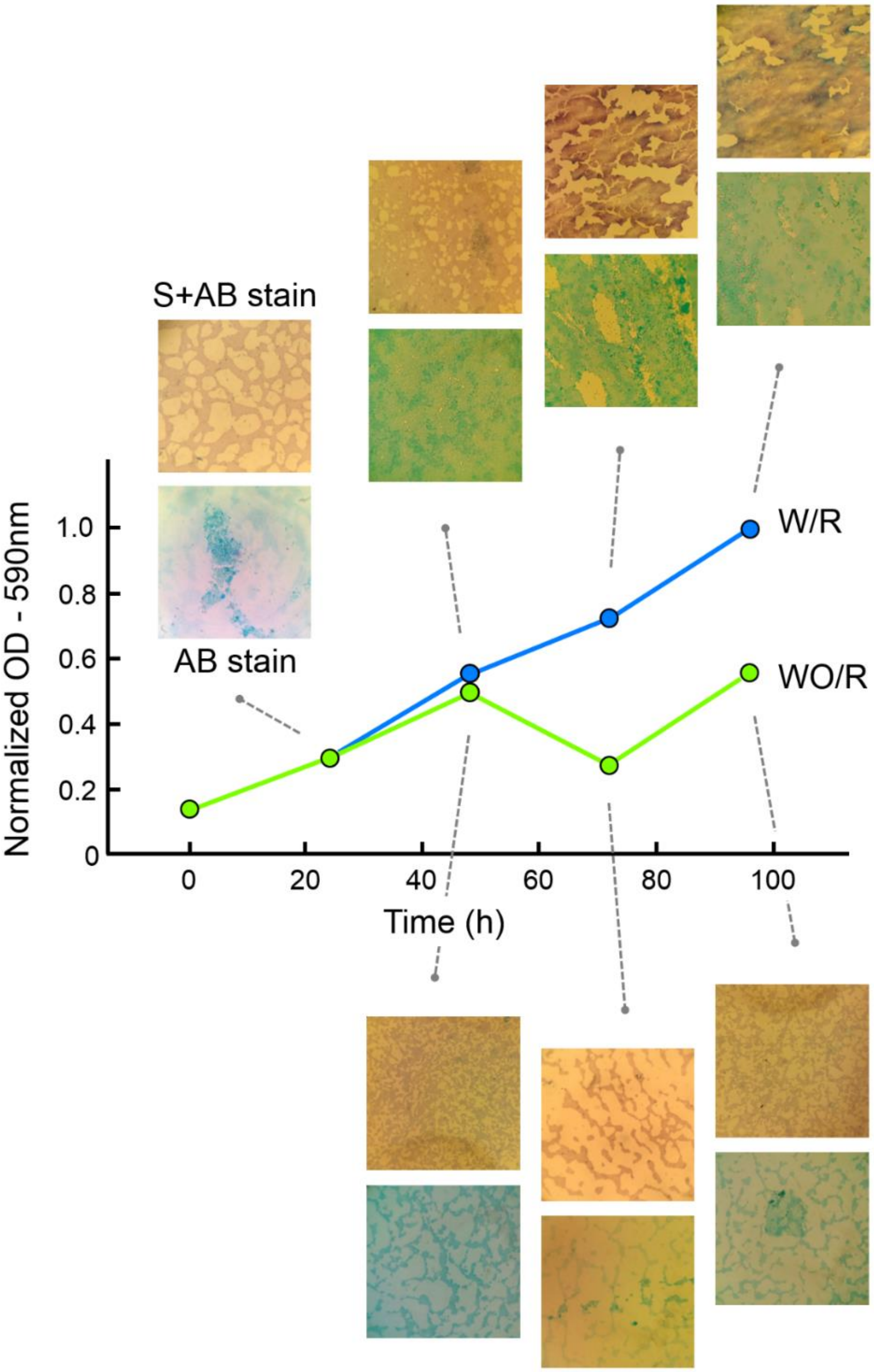

Figure S3. Optical density (OD) measurements and staining test of E. cloacae biofilms grown on glass as a function of time. The response of biofilms under no media replacement incubation conditions (WO/R) and media replacement incubation conditions (W/R) is depicted. Corresponding staining test for each time are included (WO/R and W/R).

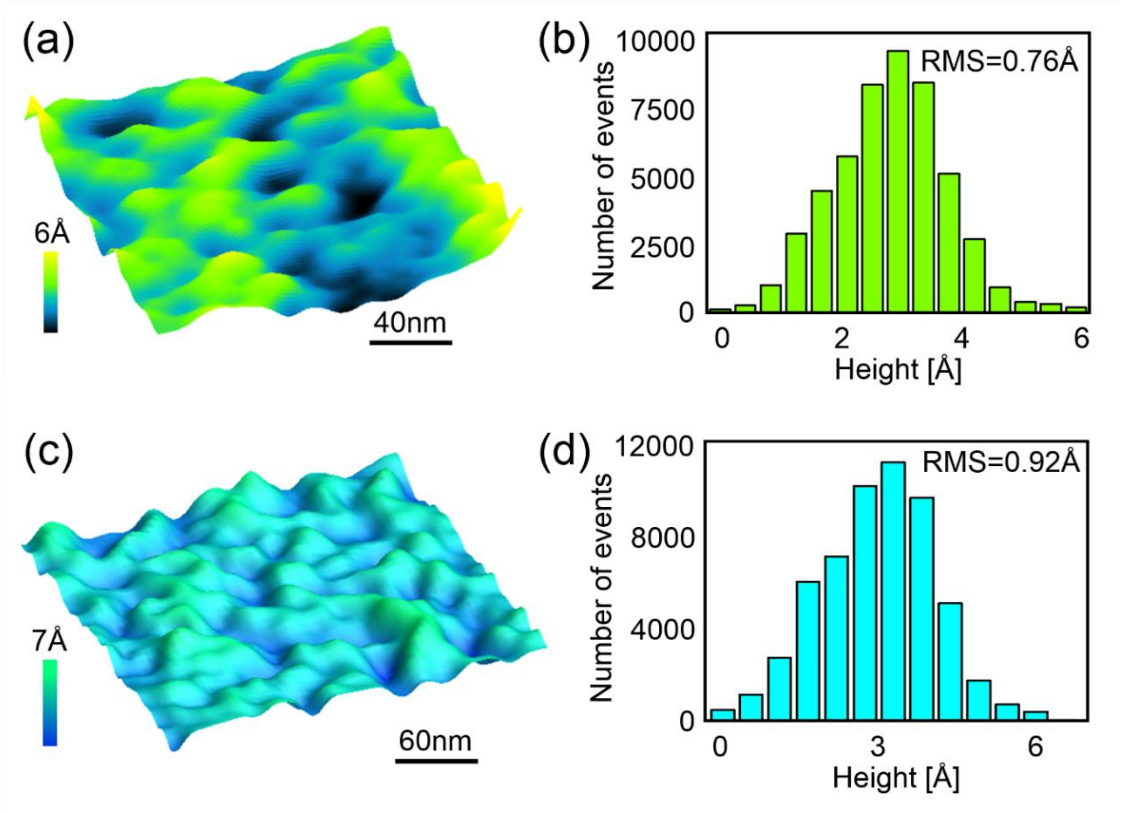

Figure S4. AFM image of graphene-coated glass sample (a) and h-BN-coated glass sample, (c) with their respective roughness analyses; (b) and (d).

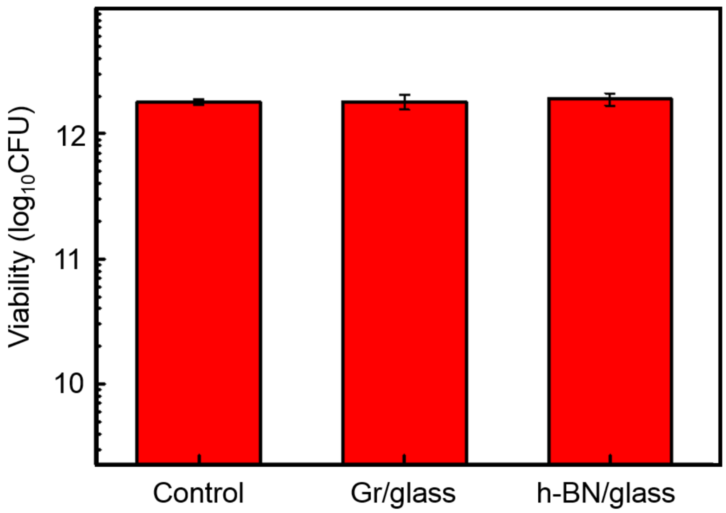

Figure S5. Cell viability of E. cloacae in planktonic state (not adhered) recovered after 48h exposure to graphene- and h-BN-coated glass.

Table S1. Morphological and biochemical test performed for strain identification of wild *Enterobacter cloacae*

Test or characteristic	Result	
	E. cloacae ATCC 13047	Wild isolate
Gram stain reaction	-	-
Cell morphology	Rod	Rod
Motility	+	+
Growth on MacConkey agar	+	+
Colonies	Pink	Pink
Precipitation of bile salts	-	-
Lactose fermentation	+	+
Glucose	+	+
Gas from glucose	+	+
Production of H_2S	-	-
Lysine decarboxylase	-	-
Ornithine descarboxylase	+	+
Simmons citrate	+	+
Indol	-	-
Oxidase	-	-
Catalase	+	+

+ = positive, - = negative
